# Predation risk alters life history strategies in an oceanic copepod

**DOI:** 10.1002/ecy.3214

**Published:** 2020-11-03

**Authors:** Kristina Øie Kvile, Dag Altin, Lotte Thommesen, Josefin Titelman

**Affiliations:** ^1^ Department of Biosciences University of Oslo PO Box 1066 Blindern Oslo 0316 Norway; ^2^ Norwegian Institute for Water Research Gaustadalléen 21 Oslo 0349 Norway; ^3^ BioTrix Trondheim NO‐7022 Norway; ^4^ Department of Biology Norwegian University of Science and Technology Trondheim NO‐7491 Norway

**Keywords:** *Calanusfinmarchicus*, chemical cues, copepod, development, growth, life history, lipid, predation risk, RNA : DNA, zooplankton

## Abstract

The ubiquitous oceanic copepod *Calanus finmarchicus* is the major link between primary producers and important fish stocks in the North Atlantic Ocean and adjacent seas. Despite over a century of research on growth and development of this key species, the effect of predation risk on these processes remains elusive. We tested how food level and chemical cues from a fish predator influence growth and development of *C. finmarchicus*, using a predator naïve laboratory population. Copepods reached adult stage earlier both in response to high food and to predator cues in our experiment. High food also increased growth and lipid accumulation. In contrast, perceived predation risk triggered reduced size and lipid fullness, indicating a decoupling of growth and development rates. Our results demonstrate that chemical predator cues can influence life history strategies in *C. finmarchicus*, and suggest that present and future patterns in oceanic zooplankton size and population dynamics may also reflect differences in predation risk.

## Introduction

Nonlethal effects of predator presence shape the behavior, morphology, and life history of prey, with potentially stronger effects on prey populations than direct consumption (Lima 1998, Preisser et al. 2005). In addition to visual, auditory, and tactile/hydrodynamic cues, prey perceive risk via chemical cues emitted by predators. Effects of chemical predator cues have been widely studied in lakes and in benthic marine invertebrates (Kats and Dill 1998). In contrast, chemical cues, and even predation risk, were traditionally considered irrelevant in the pelagic ocean (Verity and Smetacek 1996), but it is now well established that the interplay between pelagic organisms also depends on chemical signals (Pohnert et al. 2007, Heuschele and Selander 2014).

Vast, open, and dynamic, the pelagic ocean constitutes the largest living space on Earth. Pelagic copepods are omnipresent and likely the most abundant animal group on the planet, playing critical roles in marine ecosystems and biogeochemical cycles (Schminke 2007). In contrast to in air, diffusion of small molecules, such as chemical predator cues, is typically slow in water (on the order of 10^−5^cm^2^·s^−1^; Kiørboe 2008). Therefore, chemical cues are arguably less useful for assessing immediate predation risk in the ocean, as the predator may be long gone while its scent is still around. Still, plankton may assess spatiotemporal variation in the riskiness of the surroundings by the concentration of chemical cues, as a transient “landscape of fear” (Laundre et al. 2010). In fact, many pelagic organisms, including phytoplankton (Selander et al. 2011), invertebrate larvae (Forward and Rittschof 2000), jellyfish (Esser et al. 2004), and copepods (Bjærke et al. 2014, Lode et al. 2018) respond to chemical predator cues.

Still, our understanding of the roles of chemical predator cues in driving life history strategies in marine copepods remains in its infancy (Heuschele and Selander 2014). The few available studies have demonstrated effects of chemical predator cues on both behavior (van Duren and Videler 1996, Cohen and Forward 2005) and life history of copepods, including reproduction (Lasley‐Rasher and Yen 2012, Heuschele et al. 2014), growth (Bjærke et al. 2014), and development (Lode et al. 2018), suggesting its importance on a range of spatial and temporal scales. But, to date, freshwater or estuarine copepods dominate the examples (Heuschele and Selander 2014), either because chemical cues are assumed less important in the ocean or because oceanic systems are more difficult to study.

The copepod *Calanus finmarchicus* dominates zooplankton biomass in the North Atlantic Ocean and adjacent seas, is a key food web component and the main prey for several pelagic fish and early life stages of demersal fish, and probably among the world’s most well‐studied zooplankton species (reviewed in Melle et al. 2014). Still, while its growth and development have been studied for over a century (Crawshay 1913, Campbell et al. 2001, Tarrant et al. 2014), effects of chemical predator cues on these processes have to our knowledge not been tested.

*Calanus finmarchicus* spawns eggs freely in the water column that hatch and develop through six naupliar (N1–N6) and five copepodite stages (C1–C5) to adult male (C6M) or female (C6F). The life span varies greatly with the environment, from up to three generations per year in its southern range to a 1‐yr life cycle in its oceanic distribution centers and, possibly, a multi‐year life cycle in areas influenced by arctic water (reviewed in Melle et al. 2014). In experiments, development from egg to adult can be speeded up substantially with increased food or temperature (Campbell et al. 2001). The body size is also variable, with smaller size at higher temperature and larger size with increased food (Campbell et al. 2001, Wilson et al. 2015). Considering this plasticity and the strong selective force of predation (Verity and Smetacek 1996), it is conceivable that predation risk influences growth and development in *C. finmarchicus*. Although the terms growth and development are often used interchangeably, increase in body mass and life stage transition are separate processes that may respond independently to predation risk (Beckerman et al. 2007, Bjærke et al. 2014), in the same way copepod growth and development respond differently to temperature (Forster et al. 2011).

We experimentally tested the effect of chemical predator cues in combination with varying food availability on growth and development in *C. finmarchicus*, using a predator‐naïve laboratory population. Specifically, we quantified stage development, body size, lipid accumulation, carbon : nitrogen (C:N), and RNA : DNA ratios in copepods exposed to control water or to chemical cues from fish preying upon conspecifics, and to high or low food levels. Our study demonstrates for the first time how perceived predation risk alters life history strategies in this important oceanic copepod.

## Methods

### *Calanus finmarchicus* culture

All experiments were conducted with copepods from the continuous culture at NTNU SeaLab in Trondheim, Norway (Hansen et al. 2007). The culture was established from *C. finmarchicus* collected by vertical net hauls in the adjacent Trondheimsfjord in 2004, and the copepods used in our experiment have been in culture for >65 generations at an average generation length of 11–12 weeks. The culture is kept in 250‐L polystyrene tanks at 10°C and a 16:8 h light :dark cycle, with running natural seawater continuously supplied from 70 m depth in the fjord, filtered to 10 µm and at an exchange rate of 1× the tank volume daily. Copepods are continuously fed ad libitum a mixed diet of the unicellular algae *Rhodomonas baltica* and *Dunaliella tertiolecta*. To verify the genetic identity of the culture, 200 females from the broodstock producing offspring used in the experiment were identified to species using a molecular‐based protocol (Choquet et al. 2017), confirming the unique presence of *C. finmarchicus* (M. Choquet, *personal communication*).

### Experimental setup

**Fig. 1:**
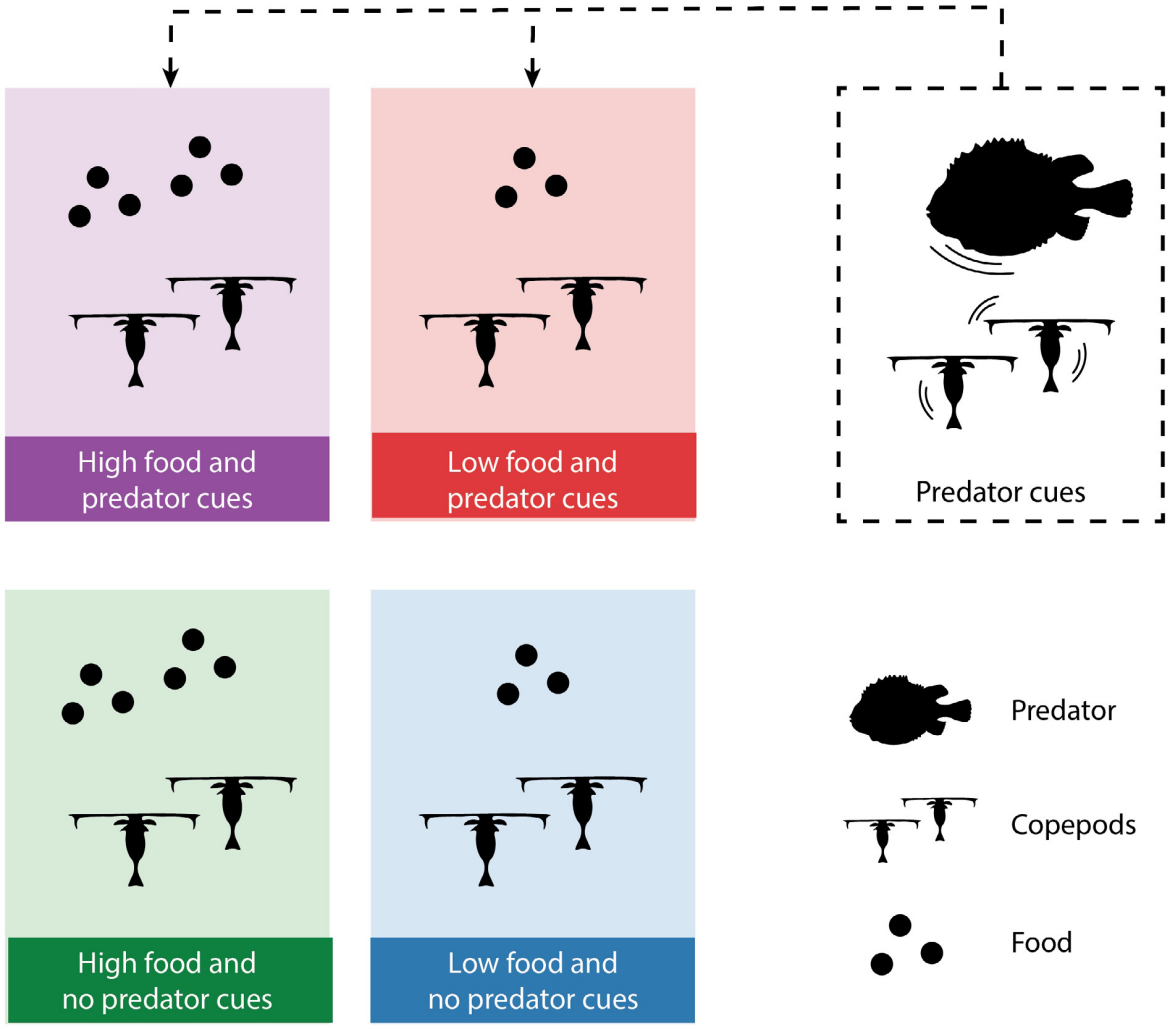
Conceptual figure of the four experimental treatments (high and low food, ±predator cues), each with three replicates. Water with chemical predator cues was continuously added to the experimental tanks from a separate tank with fish preying on copepods.

To examine the effects of predation risk and food availability on copepod life history strategies, we conducted an experiment with a 2 × 2 factorial design, with two levels of food (high, low) with and without predator cues as treatments (Fig. 1). Each treatment had three replicates, resulting in a total of 12 tanks assigned randomly to treatment. The experiment was conducted in 45‐L white HD polyethylene tanks in a temperature‐controlled room set to a fixed temperature of 10°C (±2°C in extremes, ±1°C routine) and a 16:8 h light : dark cycle, and lasted for 24 d. The tanks received a continuous supply of temperature‐controlled (9°C ± 1.5°C) natural seawater filtered to 10 µm, and of identical quality as the water used for the running cultures, at an exchange rate of 1.5× the tank volume daily (47 mL/minute). Treatments (food and predator cues) were added continuously with the filtered seawater. At regular intervals during the experiment, we sampled copepods from all tanks for image analyses of stage, size, and lipid fullness (Table 1). At specific days, all sampled copepods were further preserved for RNA : DNA ratio or carbon to nitrogen ratio (C:N) analyses.

**Table 1:** Experimental timeline.

Treatment	Day																								
	0	1	2	(3)	4	5	(6)	(7)	8	9	10	(11)	12	13	14	(15)	16	17	18	(19)	20	21	(22)	23	24
No. copepods imaged	115	71	144		145	72			73	96	180		72	120	180		71	96	176		72	114		78	95
C:N	×				×					×				×				×				×			×
RNA : DNA		×				×			×				×				×				×			×	

Notes

Copepods were sampled semi‐regularly for image analyses (number of copepods sampled and imaged indicated). At specific days, all sampled copepods were preserved and later analyzed for C:N or RNA : DNA (indicated with ×). There was no sampling on days in parentheses.

On day 0 (28 September 2019), we transferred 300 stage C4 copepodites from the culture to each experimental tank in random order by aliquoting batches of 50 animals until reaching a total of 300 per tank. To ensure that copepods were in good condition and desired developmental stage, animals were picked up with plastic spoons and kept submerged while quickly determining stage from visual inspection of size. An additional batch of 115 copepods were imaged and kept for C:N analyses. These images confirmed a dominance of C4 on day 0 (90%, mean prosome length ± SD = 1.6 ± 0.07 mm), but with 4% C3 (1.16 ± 0.04 mm) and 6% C5 (1.94 ± 0.09 mm), which we assumed to be similar across tanks.

The exposure to experimental treatments started on day 1. Phytoplankton concentrations were 200 and 90 μg C/L in high and low food treatments, respectively, which correspond to saturated and unsaturated food conditions for *C. finmarchicus* development rate (Campbell et al. 2001: Fig. 6B). Stocks of *Rhodomonas baltica* were prepared daily by diluting algae from an exponentially growing culture with filtered seawater, and added continuously to the tanks using a tubing pump. The target phytoplankton concentrations were verified using a cell counter (Multisizer3; Beckman Coulter Life Sciences, Indianapolis, Indiana, USA) and adjusted to <10% from the target if necessary. Additionally, we measured algal concentrations in the experimental tanks every 3–5 d to ensure a consistent difference between high and low food treatments throughout the experiment (mean ± SD; high food without predator cues 171 ± 26 μg C/L; high food with predator cues 181 ± 26 μg C/L; low food without predator cues 70 ± 11 μg C/L; low food with predator cues 76 ± 10 μg C/L, see Appendix S1: Fig. S1 for details).

In addition to food, the supplied water was enriched with either water with predator cues (+predator cues) or regular filtered seawater (−predator cues). We obtained the predator cues by incubating juvenile *Cyclopterus lumpus* (lumpfish) from a commercial hatchery (Tjeldbergodden Rensefisk AS) in a separate 37‐L tank with aerated filtered seawater exchanged 1.5× the tank volume daily (39 mL/min). Water from the fish tank was continuously pumped through a 20‐μm mesh and into the tanks with predator cue treatment, at a rate equivalent to 10% of the water supplied to the tanks (4.7 mL/min). On day 1, the fish tank contained 114 fish at 17 weeks post hatch with a mean mass of 0.34 g. This was reduced to 54 fish on day 12 to account for a predicted doubling of fish mass with time (O. A. Kjørsvik, *personal communication*; mean mass of removed fish 0.73 g). We fed the fish 2,200–2,700 live *C. finmarchicus* copepods (C5 or C6) from the running cultures daily, divided between four to six meals corresponding to a total of 20–50 copepods·fish^−1^·d^−1^. The predator cues were thus potentially a combination of chemicals from the fish (kairomones) and alarm cues from copepods eaten by the fish, but we assume that copepods from this dense, long‐established culture are habituated to the scent of dead conspecifics.

At each sampling event (Table 1), we collected copepods randomly from each tank with a ladle and transferred the sample to a plastic cup, keeping copepods submerged in the respective tank water. We sampled two to four tanks at a time in random order and kept samples cooled on ice. Immediately afterward, animals were picked with a wide bore pipette, placed in a drop of water, anesthetized by adding a drop of tricaine methanesulfonate solution (Finquel, 1.5 g/L seawater; Argent Laboratories, Redmond, Washington, USA), and imaged laterally using a CCD camera (Nikon DS‐Fi1/U2, Tokyo, Japan) mounted on a Leica MZAPO stereomicroscope (Leica Microsystems, Wetzlar, Germany). We picked out and anesthetized five to eight animals at a time, thus keeping exposure and handling time <5 minutes.

The experiment terminated on day 24. The remaining fish (mean mass 1.22 g) were euthanized using an overdose of tricaine methanesulfonate. A total of 23 fish died during the experiment (maximum 2 in any single day). Mortality of copepods was negligible in the experiment, i.e., nine observed copepods, corresponding to <0.5%.

### Elemental analyses

We sampled for C:N approximately every fourth day (Table 1). For stage C4, we pooled three individuals per sample to obtain sufficient material, while for C5 and C6, one individual per sample was sufficient. From day 0, we analyzed 115 copepods from the separate batch collected for starting conditions. On day 4, we sampled 12 copepods per tank (aiming for 4 × 3 C4s) and on the remaining days we sampled 8 copepods per tank. After imaging as outlined above, copepods were transferred to pre‐weighed tin capsules, dried at 60°C for 24 h, and stored in sealed boxes until analyses. C and N masses were measured using a Thermo Finnigan EA 1112 Series Flash Elemental Analyzer (Thermo Fisher Scientific, Waltham, Massachusetts, USA) and converted to individual element percentages and molar C:N.

### RNA : DNA analyses

We sampled six copepods per tank for RNA : DNA approximately every fourth day (Table 1). After imaging, copepods were placed individually in 0.5‐mL Eppendorf tubes with RNAlater (Thermo Fisher Scientific) and kept at 4°C for 24 h before storage at −20°C until analyses. RNA : DNA analyses followed the protocol by Bullejos et al. (2014). Briefly, we obtained estimates of individual RNA and DNA (μg) using a microplate fluorometric high‐range RiboGreen assay (Quant‐iT RiboGreen RNA Assay Kit; Thermo Fisher Scientific) after extraction in 1% sarcosyl (prepared with *N*‐Lauroylsarcosine and Tris‐EDTA buffer; Merck Life Science, Darmstadt, Germany) and RNase digestion (RNase DNasefree, working solution 5 μg/mL, Merck Life Science). We performed fluorescence measurements using a BioTek Synergy Mx Microplate Reader, and converted measurements into individual RNA and DNA content (and thus RNA : DNA) using standard curves (16S and 23S RNA from *Escherichia coli*, RiboGreen RNA Assay Kit, Thermo Fisher Scientific; DNA from calf thymus, Merck Life Science).

### Image analyses

We used the software ImageJ and a drawing tablet (Wacom Cintiq 12wx; Wacom, Saitama, Japan) to analyze images of all sampled copepods, calibrating the pixel‐to‐mm ratio using a stage micrometer imaged at the respective magnifications. The prosome and lipid sac were outlined manually and their two‐dimensional projected areas (hereafter areas) quantified in ImageJ. We estimated lipid fullness as the percentage of the prosome area comprised by the lipid sac area.

### Statistical analyses

All statistical analyses were performed in R (version 3.3.2; R Core Team 2016) using the mgcv library for GAMs (Wood 2006). Data and code to run the analyses are available; see *Data Availability* statement. We formulated generalized additive mixed models (GAMMs) to assess effects of treatments on copepod development and size, lipid fullness, C:N, and RNA : DNA throughout the experiment. This allowed us to use smooth functions to represent the effect of sampling day, since we assumed the response variables could change nonlinearly with time. First, we investigated effects on development time

(1) $$S∼\beta +F+P+g\left(D\times F\right)+g\;\left(D\times P\right)+g\;\left(T\right)+\varepsilon$$

here, *S* is the mean developmental stage of the sampled copepods per day and tank (C4 = 4, C5 = 5, C6F/C6M = 6), β the intercept, *F* a factor variable of food level (high/low), and *P* is a factor level of predator cues (±predator cues). g(*D* × *F*) and g(*D* × *P*) are interactions between a smooth function of sampling day and food or predation, respectively; i.e., the smooth effect of sampling day is allowed to differ between high and low food level, and with and without predator cue. The term g(*T*) is a random effect of experimental tank specified using the flag bs = re, which produces a random coefficient for each level of the factor, and ε is a normally distributed error term. The smooth functions of day had maximally four knots, i.e., 3 degrees of freedom. For model diagnostic plots, see Appendix S1: Fig. S2.

Second, we investigated effects of treatments on prosome area, lipid fullness, C:N, and RNA : DNA. We first tested for significant differences in the variables between stages using the nonparametric, two‐sided, Wilcoxon rank sum test (the Shapiro‐Wilk test indicated that data were not always normally distributed) and subsequently fitted a GAMM per stage

(2) $$Y∼\beta +F+P+\;g\left(D\times F\right)+g\;\left(D\times P\right)+g\;\left(T\right)+\varepsilon$$

here, *Y* is the observations of the response variable for the given stage, and other model terms correspond to Eq. 1. We assumed a normal error distribution in all models, which was reasonable for most variables and stages, except for RNA : DNA (Appendix S1: Figs. S3–S7). Natural‐log‐transforming the RNA : DNA data improved model diagnostics, and we therefore present results with log‐transformed data. *P* values for the different covariates were extracted from the summary of the fitted GAMs, and we used a significance level of 0.05. To compare relative effects of food level and predator cue on data expressed in different units, we standardized each of the response variables in Eq. 2 as unit of standard deviation when calculating coefficient estimates of the factor variables, i.e., coefficient estimates were extracted from models fitted to data standardized per stage, by first subtracting the stage‐specific mean and then dividing by the stage‐specific standard deviation of the response variable in question.

## Results

### Development

**Fig. 2:**
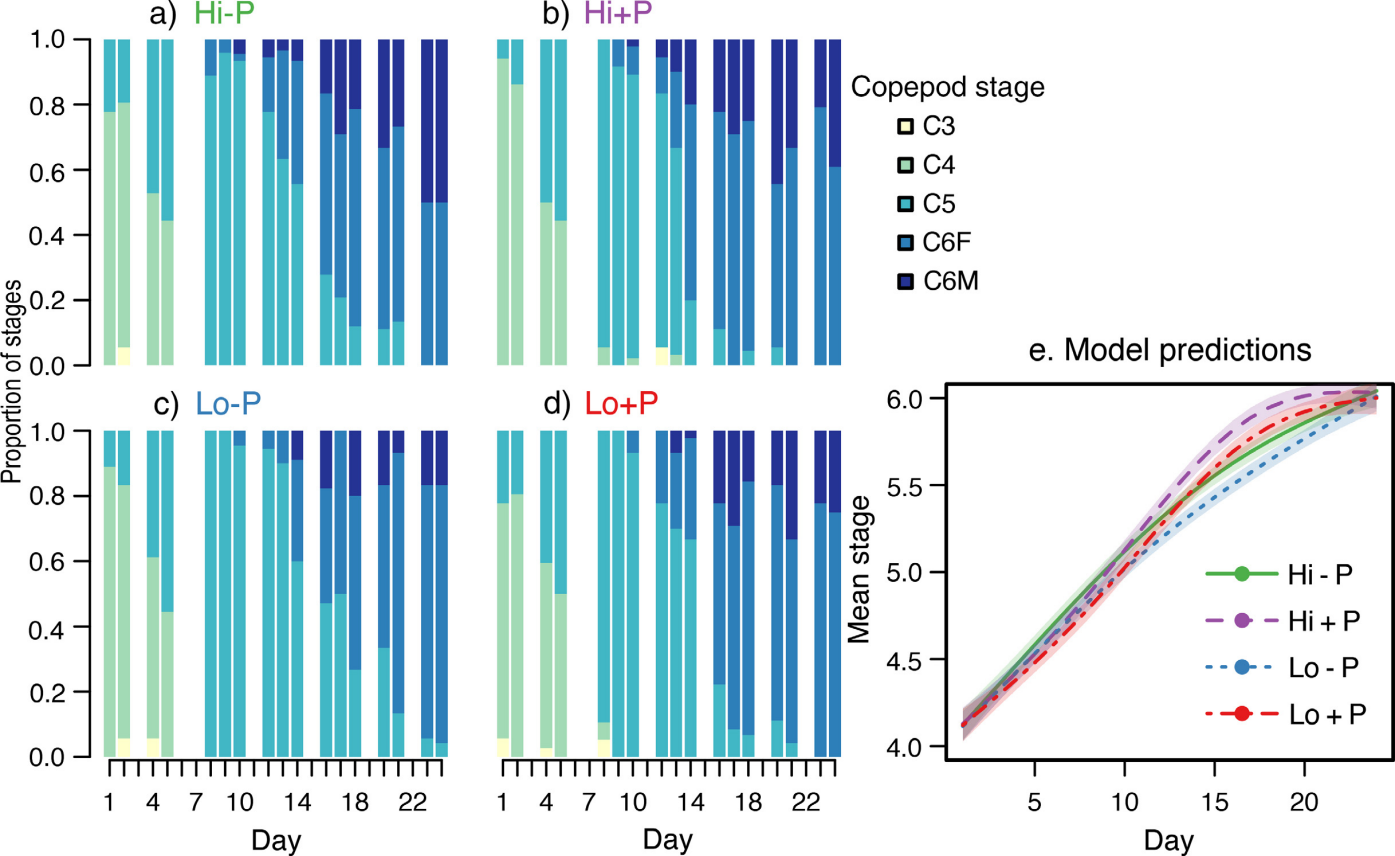
*Calanus finmarchicus* development stage by day. (a–d) Fraction of sampled copepods in different developmental stages (color scale) per sampling day and treatment. (e) Predicted mean stage per day and treatment (colored lines, corresponding to text color in panels a–d) from the statistical model (Eq. 1), with 95% confidence interval of the prediction (shaded areas). Hi − P, high food and no predator cues; Hi + P, high food and predator cues; Lo − P, low food and no predator cues; Lo + P, low food and predator cues. F, female; M, male.

Food and predator cues significantly affected stage development, and effects varied with time as indicated by the interactions between day and food or predator cues (Fig. 2, Table 2). Generally, adults appeared earlier both in high food treatments and with predator cues (Fig. 2a–d). Predictions from the statistical model (Eq. 1) indicated that from around day 14, the development stage was more advanced in treatments with predator cues than without predator cues, regardless of food availability (Fig. 2e). We also tested to include an interaction effect of food and predator cues in the model, but it was nonsignificant.

**Table 2:** Adjusted *R* ^2^, coefficients estimates for parametric factor variables, and *P* values for all model terms in the statistical models of observed variation in *Calanus finmarchicus* mean development stage (Eq. 1), and prosome area (mm^2^), lipid fullness (lipid sac area as percentage of prosome area), C:N, and RNA : DNA ratio (natural log‐transformed) per developmental stage (Eq. 2).

Response and copepod stage		Coefficient estimates		Treatment and tank *P*			Day × Treatment *P*	Day × Treatment *P*	Day × Treatment *P*	Day × Treatment *P*
	*R* ^2^	Food	Predator cue	Food	Predator cue	Tank	Low food	High food	No predator cue	Predator cue
Development	0.95	0.08	0.06	**<0.01**	**<0.01**	0.46	**<0.01**	**<0.01**	**<0.01**	**<0.01**
Prosome area										
C4	0.12	0.00	−0.23	0.97	0.08	0.24	0.04	**<0.01**	0.68	0.16
C5	0.49	0.60	−0.68	**<0.01**	**<0.01**	**<0.01**	0.10	0.18	**<0.01**	**<0.01**
C6F	0.54	0.64	−1.24	**<0.01**	**<0.01**	**<0.01**	**0.01**	**0.02**	**<0.01**	**0.03**
C6M	0.37	0.66	−0.94	**<0.01**	**<0.01**	0.10	**0.04**	0.36	**0.03**	0.95
Lipid fullness										
C4	0.50	0.36	−0.42	**<0.01**	**<0.01**	0.71	**0.03**	0.13	**<0.01**	**<0.01**
C5	0.59	0.55	−0.37	**<0.01**	**<0.01**	**<0.01**	**0.02**	**<0.01**	**<0.01**	**0.04**
C6F	0.50	0.82	−1.10	**<0.01**	**<0.01**	**<0.01**	0.28	**0.03**	**0.02**	0.08
C6M	0.31	0.09	−0.70	0.56	**<0.01**	0.12	0.77	**<0.01**	0.10	0.25
C:N										
C5	0.63	0.33	−0.36	**0.01**	**<0.01**	**0.01**	**<0.01**	0.39	**<0.01**	**<0.01**
C6F	0.35	0.46	−1.04	**0.01**	**<0.01**	**0.03**	0.29	0.55	0.86	0.21
C6M	0.48	‐0.15	−0.78	0.73	0.07	**<0.01**	0.53	**<0.01**	**0.03**	0.53
ln(RNA : DNA)										
C5	0.15	0.21	0.00	0.12	0.98	0.68	0.10	0.06	**0.01**	0.12
C6F	0.23	0.52	0.53	**<0.01**	**<0.01**	0.82	0.57	0.28	**0.02**	0.21
C6M	0.48	0.17	−0.13	0.57	0.65	0.08	0.10	0.53	0.21	0.31

Notes

Coefficient estimates indicate the mean predicted change in the response variable when the predictor variable moves from low to high (food level) or from absence to presence (predator cue). For prosome area, lipid fullness, C:N, and RNA : DNA, coefficient estimates are reported as stage‐specific standard deviations. Interactions between day and food or predator cue are formulated as different smooth effects of day under different factor levels. *P* < 0.05 are shown in boldface type. M, male; F, female.

### Growth

**Fig. 3:**
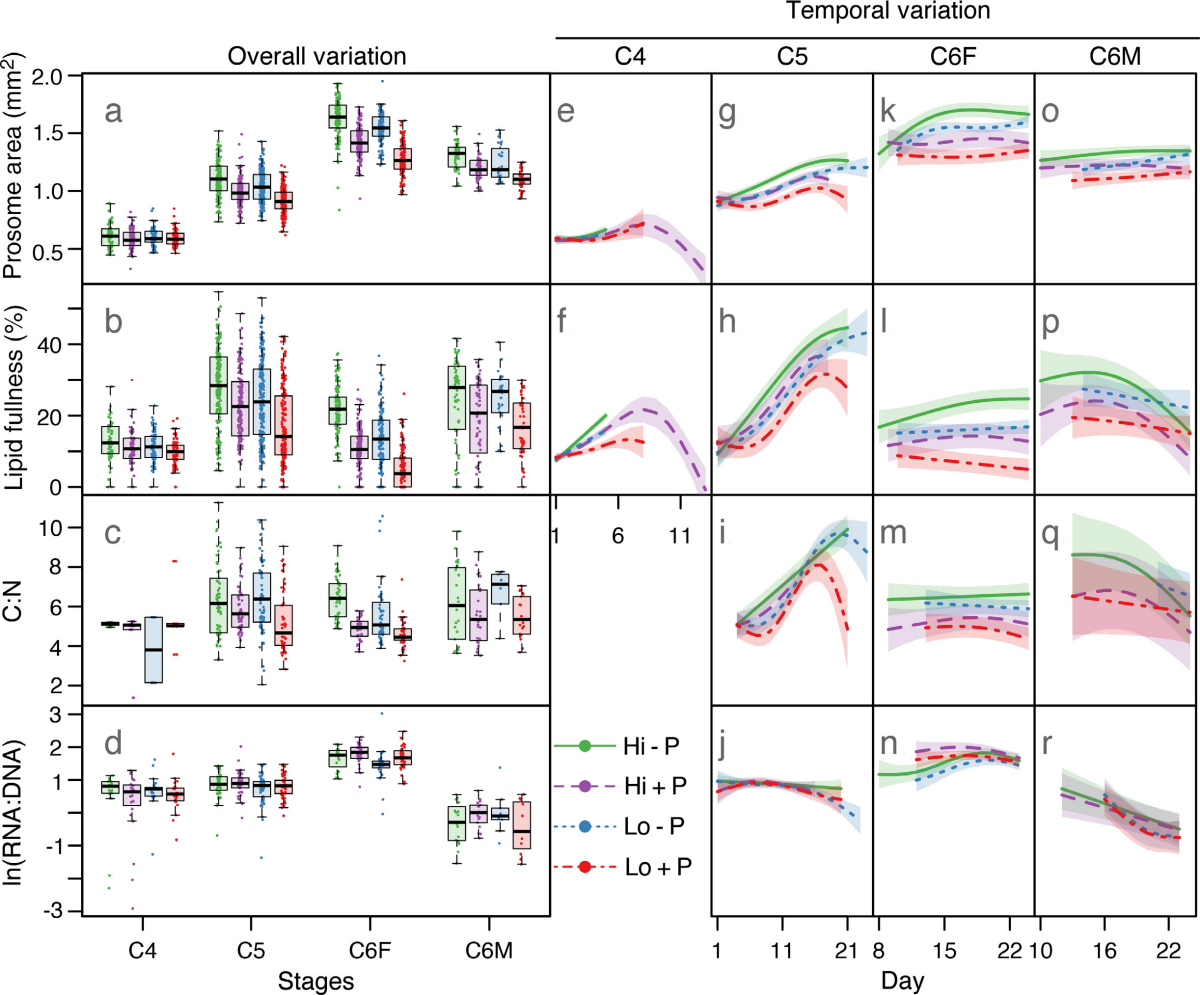
*Calanus finmarchicus*  two‐dimensional prosome area (mm^2^), lipid fullness (lipid sac area as percentage of prosome area), C:N, and RNA:DNA ratio (natural‐log‐transformed) per developmental stage and treatment. (a–d) Observations per stage and treatment (points) with overlain box plot of the median (line), the interquartile range (box), and 1.5 × the interquartile range (whiskers) of the data. (e–r) Predicted temporal variation per stage and treatment (lines) from the statistical model (Eq. 2), with 95% confidence interval of the prediction (shaded areas). Predictions are plotted for days with available data for the given stage and treatment (C:N and RNA : DNA data were not sufficient to fit Eq. 2 for C4). The *x*‐axis differs between stages. Hi − P, high food and no predator cues; Hi + P, high food and predator cues; Lo − P, low food and no predator cues; Lo + P, low food and predator cues.

Food and predator cues had opposite effects on size, lipid fullness and C:N. While food significantly increased prosome area (C5, C6F, and C6M; Fig. 3a), lipid fullness (C4, C5, and C6F; Fig. 3b) and C:N (C5 and C6F; Fig. 3c), predator cues had a negative, and often stronger, effect than food on the same end points (Table 2). RNA : DNA in C6F increased with both food and predator cues, but was similar between treatments in other stages (Fig. 3d). Again, we tested the inclusion of an interaction effect of food and predator cue, but this interaction was always nonsignificant. In general across treatments, prosome area increased from C4 to C6F, with C6M being between C5 and C6F; lipid fullness and C:N was higher in C5 and C6M compared to C4 and C6F; and RNA : DNA was highest in C6F and lowest in C6M (Fig. 3a–d). However, there were differences between treatments, for example, lipid fullness was higher in C6F than C4 in treatments without predator cues (with high or low food), and lower in C6F than C4 in the low food and predator cue treatment (Fig. 3b). The effect estimates of food level and predator cue, calculated using data standardized per stage, underscore that the largest differences between treatments occurred in C6F with and without predator cues; and the presence of predator cues had strongest effect on prosome area, thereafter lipid fullness and C:N, and a relatively weaker effect on RNA : DNA (coefficient estimates; Table 2).

By including interactions between day and treatments, we could compare temporal patterns in response variables (Fig. 3e‐r). Observations in time stem from different copepods and not the same individuals observed repeatedly. Therefore, the observed trends can be due to both developmental changes from the initiation to the end of a stage, or to differences between individuals that happened to reach a given stage relatively early (or late) in the experiment. There were significant interaction effects between day and predator cues on prosome area and lipid fullness in C5 (Fig. 3g,h) and C6F (Fig. 3k,l), suggesting faster growth and lipid accumulation in treatments without predator cues during the first two weeks. In C5, lipid fullness clearly increased with time (Fig. 3h), while in C6F, lipid fullness tended to increase with time in the high food and no predator cue treatment, decrease in the low food and predator cue treatment, while remaining relatively stable in the other two treatments (Fig. 3l). In C6M, a decrease in lipid fullness after the first two weeks was predicted to be steeper in treatments with high food (Fig. 3p). C:N also increased with time in C5, and the results suggested an earlier increase in C:N with high food and a later but steeper increase with low food, ultimately reaching highest levels in treatments without predator cues (Fig. 3i). In C6M, temporal patterns in C:N resembled lipid fullness (Fig. 3q), while in C6F, there was no significant change in C:N with time (Fig. 3m, Table 2). RNA : DNA in C6F was first highest in treatments with predator cues, but a decrease after day 19 that did not occur without predator cues led to similar levels toward the end of the experiment (Fig. 3n). There was not sufficient data to describe temporal variation in C:N and RNA : DNA for C4.

To ease interpretation of C:N and RNA : DNA results, we performed supplementary analyses of C, N, DNA, and RNA as individual mass (μg) and percentage of body mass (Appendix S1: Fig. S9 and Table S1). The results suggest that the negative effect of predator cues on C:N in C5 and C6F was driven by decreased C relative to N, and the positive effect of food by a stronger increase in C compared to N. In C5, RNA : DNA was not related to predator cues, but weakly related to food (*P* = 0.13, Table 2), and percent RNA was positively related to food (*P* < 0.05, Appendix S1: Table S1). In C6F, DNA (μg) was negatively related to predator cues but unrelated to food, while RNA (μg) was positively related to food but unrelated to predator cues (Appendix S1: Table S1). This suggests that the positive effect of food on RNA : DNA in C6F was driven by increased RNA, and the positive effect of predator cues on RNA : DNA by reduced DNA. Percent DNA did not differ significantly between treatments in any stage.

## Discussion

Mirroring the traditional bottom‐up perspective in marine pelagic ecology (Verity and Smetacek 1996) and the key role of *Calanus* copepods in Atlantic and Arctic food webs (Falk‐Petersen et al. 2009), a myriad of studies exist on effects of temperature and food on *C*. *finmarchicus* growth and development (Crawshay 1913, Clarke and Bonnet 1939, Corkett et al. 1986, Campbell et al. 2001, Tarrant et al. 2014). In contrast and for the first time, we show how perceived predation risk alters investments in development and growth in this important species. Perceived predation risk led to faster development but smaller size and reduced lipid accumulation. Our results thus suggest that top‐down forces have the potential for shaping life history in *C*. *finmarchicus*.

*Calanus finmarchicus* is a major prey for planktivorous fish such as *Clupea harengus* (herring) and *Scomber scombrus* (mackerel; Prokopchuk and Sentyabov 2006,). The migration patterns and life histories of these fish are tightly coupled to *C*. *finmarchicus* life history events such as diapause and reproduction (Varpe and Fiksen 2010, Opdal and Vikebø 2016). Furthermore, copepod size affects detectability and encounter by visual predators and, also via energy content, the growth rates of planktivorous fish (van Deurs et al. 2015). Climate‐driven changes in sea ice cover or water clarity can in turn impact the visual search efficiency of planktivorous fish and thereby the size‐dependent predation pressure on copepods (Dupont and Aksnes 2013, Langbehn and Varpe 2017). Thus, the size and life history of *Calanus* copepods are both critical for, and impacted by, predator–prey interactions with fish, and these effects are highly relevant in the light of climate change (Kaartvedt and Titelman 2018).

A large body of literature exists on the role of chemical cues in predator–prey interactions in pelagic and benthic freshwater invertebrates, as well as in marine benthic invertebrates (Kats and Dill 1998). In contrast, the role of chemical predator cues in the pelagic ocean remains less explored (Heuschele and Selander 2014). Prey may respond to predation risk by altering behavior, morphology, or life history. The dominant zooplankters in lakes, cladocerans, can develop protective spines, helmets or other morphological defenses in the presence of chemical predator cues (Tollrian and Dodson 1999). In contrast, pelagic copepods have relatively similar and fixed body shapes, possibly because protective structures are ineffective against planktivorous marine fish (Verity and Smetacek 1996). Moreover, copepod form and function are well adapted for escape. Many copepods respond to fluid signals with extremely rapid and powerful escape jumps, enabled by separate propulsion systems for regular swimming and for escape (Kiørboe 2011).

Still, when faced with planktivorous fish, an even more efficient strategy is to seek refuge in the darker, deeper layer through diel or seasonal vertical migrations (Pasternak et al. 2006). Diel vertical migration (DVM), hiding at depth during day and ascending to feed at night, is a common predator avoidance strategy in zooplankton (Hays 2003). Although our experiment did not enable proper DVM, one could expect reduced foraging in response to predation risk, as active copepods or copepods with visible gut contents are more conspicuous (van Duren and Videler 1996, Tsuda et al. 1998). Reduced foraging under predation risk is common in both aquatic and terrestrial animals (Benard 2004). In line with theory on behavioral responses to non‐size‐selective predation (Abrams and Rowe 1996), reduced foraging typically leads to reduced growth and smaller size at maturity, but is often associated with delayed development (Benard 2004, Beckerman et al. 2007). Faster development in the predator cue treatment therefore suggests that altered feeding behavior was not the main response in our experiment. Still, reduced foraging or increased energy use due to stress in copepods exposed to constant predation risk may have contributed to reduced size and lipid accumulation (Slos and Stoks 2008).

While the link between predation risk and DVM is well established, the mechanisms behind the timing of *Calanus* copepods’ seasonal vertical migrations to diapause at hundreds of meters to >1,000 m depth remain elusive (Johnson et al. 2008, Melle et al. 2014). It has been proposed that predation risk may trigger diapause (Pasternak et al. 2006, Ji 2011), and we could accordingly expect that predator cues would trigger lipid accumulation in preparation for diapause and halted development in C5, the main diapausing stage in nature (Falk‐Petersen et al. 2009). Instead, lipid accumulation was lower and development to adult faster with predator cues (Figs. 2, 3). Given that our experimental environment lacked the vertical structure of a several hundred meters deep water column and that the experimental population stems from a non‐diapausing culture (Tarrant et al. 2014), it is not surprising that diapause was not induced. While diapause likely is a response to a combination of environmental cues and internal lipid content (Häfker et al. 2018), it is clear that perceived predation risk may alter developmental patterns and energy storage in this oceanic species, and one may speculate that this could affect diapause in nature.

Confirming ecological theory (Ball and Baker 1996), experimental studies have demonstrated that size‐selective predation risk can drive life history traits in similar directions as size‐selective mortality, for example, selection for large prey should trigger earlier maturation at smaller size as prey prioritize reproduction over growth (Riessen 1999, Beckerman et al. 2007). Accordingly, we observed faster development and smaller size in predator cue treatments. Increased food also speeded up development, however, food and predator cues affected size and lipid storage in opposite directions. RNA : DNA and percent RNA, common proxies for copepod growth rate (Gorokhova 2003), were positively associated to food in C5, but were not affected by predator cues (Fig. 3, Table 2; Appendix S1: Table S1). These differing growth responses suggest a decoupling of growth and development rates with predation risk; i.e., while altered energy intake in response to food level was reflected in both growth and development rates, predation risk likely triggered a physiological shift in development rate resulting in less time and resources for growth (Beckerman et al. 2007).

Subsequently, ecological theory predicts that selection for small prey should trigger increased growth to escape the predation window (Riessen 1999, Beckerman et al. 2007) or, in animals that change habitat as adults, earlier maturation (Benard 2004). In general, copepods can increase size through intra‐stage growth (size at stage) and by molting to a more advanced development stage. As perception, motility, and escape behavior develop over ontogeny (Kiørboe et al. 1999, Titelman and Kiørboe 2003), it might be more beneficial to invest in molting than intra‐stage growth when risk is high. Thus, although our results align with predicted responses to selection for large prey, it is conceivable that selection for small prey could similarly trigger faster development. We therefore encourage future studies that compare responses to predators with different selectivity.

From an evolutionary perspective, individual success depends on long‐term reproductive output. While shorter generation time increases fitness and reduces the chance of dying before reproducing, fecundity is often positively correlated with size, creating a trade‐off between growth and development (Stearns and Koella 1986). In income breeders such as *C. finmarchicus*, egg production relates to ambient feeding conditions, but possibly also to feeding history via positive effects of size and lipid stores on maturation and egg production (Richardson et al. 1999, Head et al. 2013). RNA : DNA reflects egg production rate in marine copepods (Gorokhova 2003), and as did Wagner et al. (2001), we observed an increase in RNA : DNA from C5 to C6F, likely related to egg production, and a positive effect of food on RNA content and RNA:DNA in C6F, possibly related in part to increased female size. In contrast, we did not detect effects of predation risk on reproduction as indicated by RNA : DNA, since while RNA : DNA in C6F increased with predator cues, this was driven by reduced DNA, not increased RNA. The decrease in DNA may be linked to reduced growth via lower cell numbers, or, if cell number is constant within stages, stress‐induced cell death (discussed in Speekmann et al. 2007) or reduced cell specific DNA (discussed in Wagner et al. 2001) with predation risk.

One could expect that copepods’ ability to respond to predator cues would be reduced or lost after >65 generations in culture. Culture conditions with ample food select for continuous feeding and fast growth, and thereby against behaviors that reduce growth, such as diel feeding cycles (Tiselius et al. 1995, Olivares et al. 2020) and potentially predator avoidance. Moreover, the culture population likely differs from wild populations due to founder effects, genetic drift and inbreeding, which typically result in loss of genetic variation (Futuyma 2005). To reduce the initial bottleneck effect and subsequent genetic drift and inbreeding, the culture population has been kept as large as feasible, in 5–10 250‐L tanks of 10–50,000 individuals per tank. The fact that we observed effects of predator cues on growth and development may suggest that these are fundamental and well‐preserved responses. Alternatively, one may hypothesize that cultured copepods respond more strongly than wild animals, as the latter are continuously exposed to a range of predator cues. Similarly, one could expect copepods to become habituated during the course of the experiment (Holomuzki and Hatchett 1994). In sum, while the absolute effects reported here may not translate directly to wild populations, our study clearly indicates that predation risk influences growth and development rates in oceanic copepods.

Reduced body size has been proposed as “the third universal ecological response to global warming” across aquatic and terrestrial systems (Daufresne et al. 2009, Sheridan and Bickford 2011). In the ocean, increased temperatures and growing season length with climate change favor smaller, less lipid‐rich copepods with shorter generation time, both through changes in community composition and in intraspecific growth and development rates (Forster et al. 2011, Rice et al. 2015). Higher population turnover rate may increase the energy available for higher trophic levels (Renaud et al. 2018), nevertheless, the ongoing borealization of Arctic ecosystems appears to favor southern consumers at the expense of arctic species (Fossheim et al. 2015). Our results demonstrate that in addition to temperature and food, predation risk drives life history strategies in a highly abundant oceanic copepod. Thus, observed patterns in size and development rate in *C. finmarchicus*, and potentially other *Calanus* copepods, may also reflect differences in predation risk. We may thus speculate that climate‐driven distribution shifts in both copepods and planktivorous fish will alter the growth and development rate, and thus population dynamics, of oceanic copepods, with important consequences for marine ecosystems.

## Supporting information

Appendix S1

## Data Availability

Data are available on Zenodo: http://doi.org/10.5281/zenodo.4048277
