# Supplementary material for: Predation risk alters life history strategies in an oceanic copepod
Source: Ecology. 2020 Nov 3;102(1):e03214. doi: 10.1002/ecy.3214 (PMC7816270; doi:10.1002/ecy.3214)
Supplement: Supplementary file 1 — Appendix S1 [file ECY-102-e03214-s001.pdf]

Supporting Information. Kvile, K.Ø., D. Altin, L.Thommesen, and J. Titelman. 2020. Predation risk alters life history strategies in an oceanic copepod. Ecology.

Appendix S1

Phytoplankton concentrations in the experiment 2

Fig. S1 2

Statistical model diagnostics 3

Fig. S2 3

Fig. S3 4

Fig. S4 5

Fig. S5 5

Fig. S6 6

Fig. S7 6

Effects on C, N, DNA and RNA as µg or % of body weight 7

Fig. S8 7

Fig. S9 9

Table S1 10

Phytoplankton concentrations in the experiment

Copepods were fed the unicellular algae *Rhodomonas baltica* at levels of 200 and 90 µg C L^{-1} in the high and low food treatments, respectively. We used a generalized additive mixed model (GAMM) to quantify observed variation in phytoplankton concentration (measured at days 3, 8, 11, 15, 19 and 22 of the experiment):

$$C \sim \beta + g(D) + F + P + g(T) + \varepsilon \qquad \text{(S1)}$$

C is phytoplankton concentration (µg C L^{-1}) per day and tank, β the intercept and g(D) a smooth function of sampling day (with maximally 4 knots, i.e. 3 degrees of freedom). F and P are factor variables of food (high/low) and predator cue (presence/absence), g(T) a random effect of experimental tank and ε a normally distributed error term.

The results of the statistical model showed a consistent difference between high and low food treatments (Fig. S1, P of factor effect of food < 0.01). Phytoplankton concentrations also tended to be higher in treatments with predator cue (P of factor effect of predation = 0.01), and increased in all treatments between days 10 and 15 (P of the smooth effect of day < 0.01). The random effect of tank was non-significant (P = 0.8). The adjusted R^2 of Eq. S1 was 0.94.

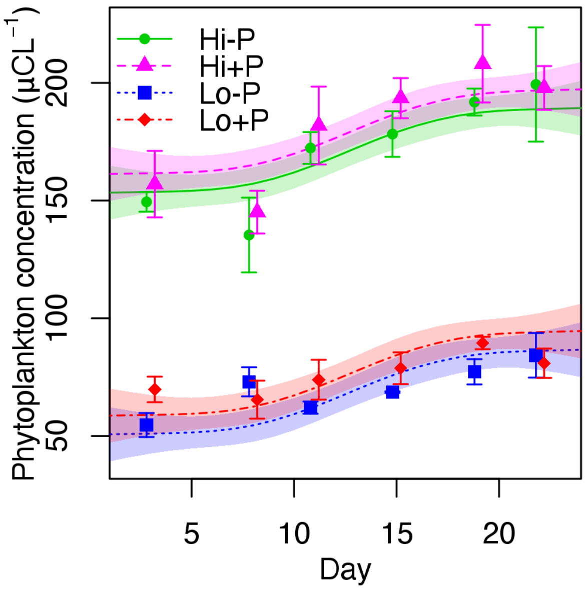

Fig. S1. Phytoplankton concentration (µg C L^{-1}) per day and treatment. Symbols and vertical lines: mean ± SD of the observations per treatment and day; lines and shaded areas: statistical model predictions with 95% confidence intervals per day and treatment (Eq. S1). Hi-P = High food and no predator cue; Hi+P = High food and predator cue; Lo-P = Low food and no predator cue; Lo+P = Low food and predator cue.

Statistical model diagnostics

To examine the fit of the statistical models (Eq. 1 and Eq. 2, main text) to the model assumptions, we plotted a range of model diagnostic plots. Below are plots for each of the following response variables: mean developmental stage (Fig. S2), prosome area (Fig. S3), lipid fullness (Fig. S4), C:N ratio (Fig. S5), RNA:DNA ratio (Fig. S6, not used in further analyses) and natural log-transformed RNA:DNA ratio (Fig. S7). For Eq. 2, models were fitted per developmental stage separately (panel rows in Fig. S3-Fig. S7). The diagnostic plots include: a) histogram of the data distribution; b) Q-Q plot (quantile-quantile plot); c) histogram of the model residuals; d) model residuals (y axis) against linear predictor (x axis); e) response (y axis) against fitted values (x axis).

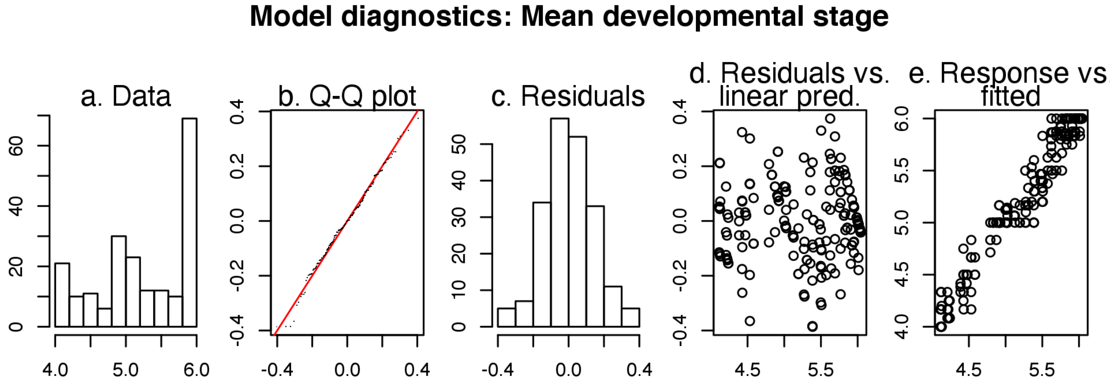

Fig. S2

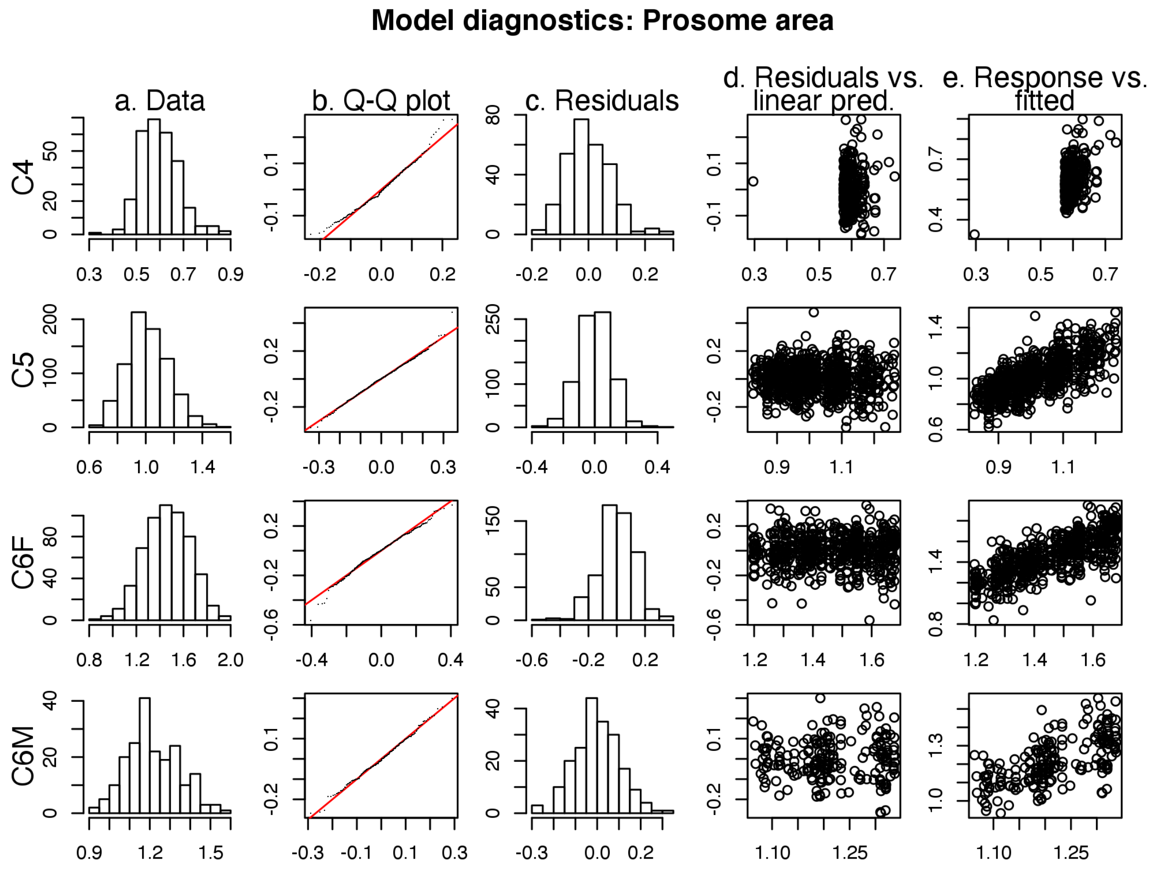
Model diagnostics: Prosome area
a. Data
b. Q-Q plot
c. Residuals
d. Residuals vs. linear pred.
e. Response vs. fitted
C4
C5
C6F
C6M

Fig. S3

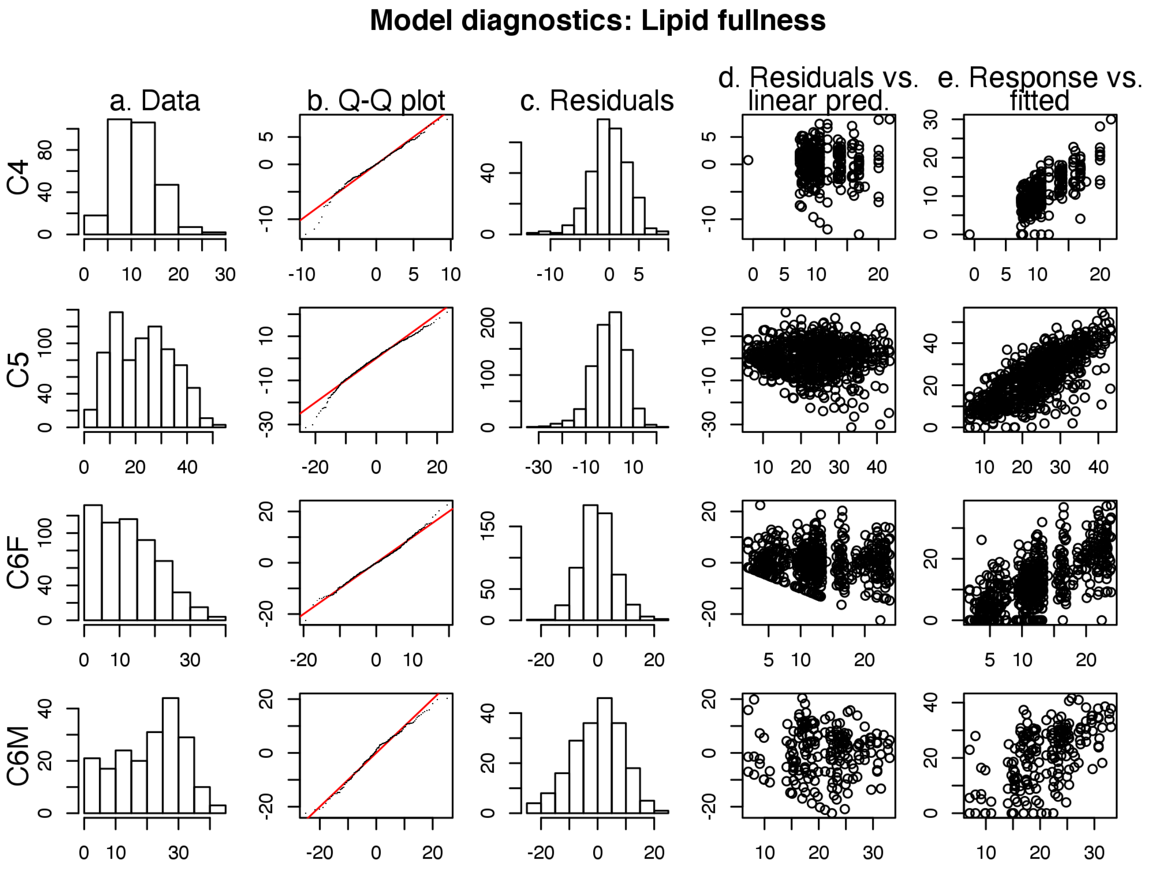
Model diagnostics: Lipid fullness
a. Data
b. Q-Q plot
c. Residuals
d. Residuals vs. linear pred.
e. Response vs. fitted
C4
C5
C6F
C6M

Fig. S4

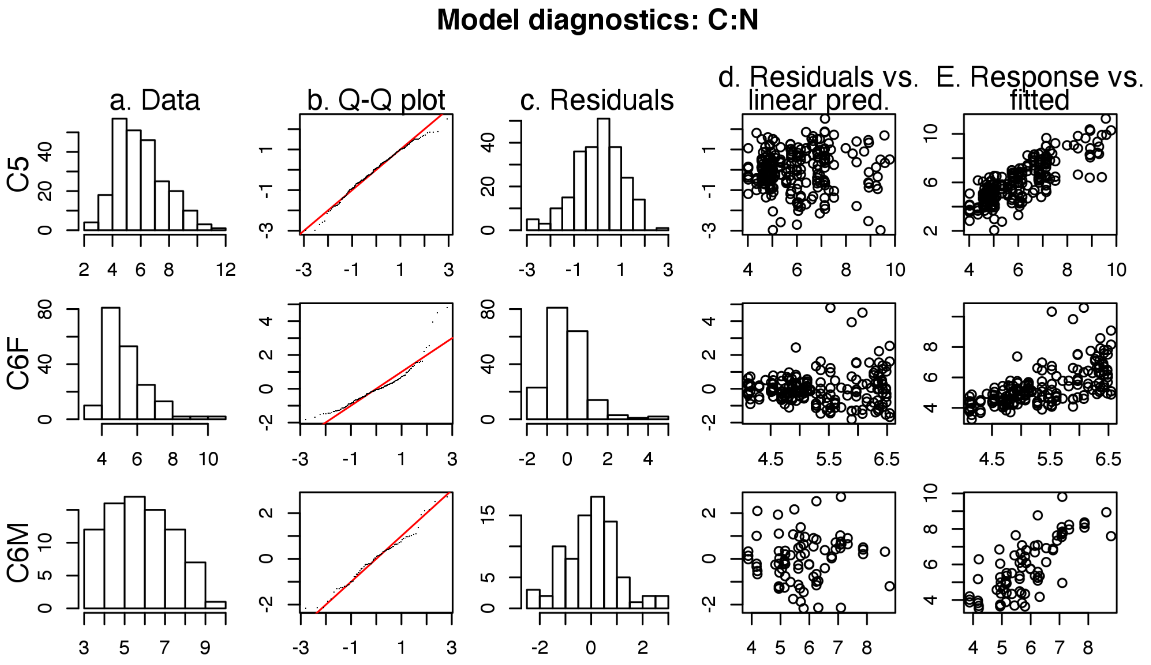
Model diagnostics: C:N
a. Data
b. Q-Q plot
c. Residuals
d. Residuals vs. linear pred.
E. Response vs. fitted
C5
C6F
C6M

Fig. S5

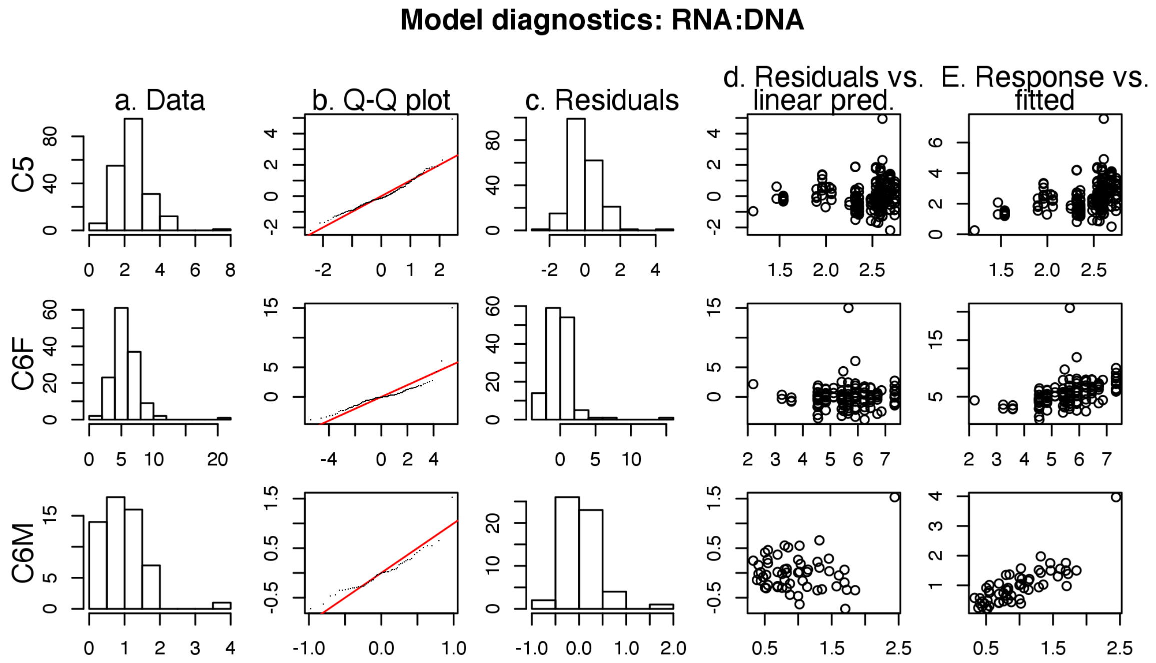
Model diagnostics: RNA:DNA
a. Data
b. Q-Q plot
c. Residuals
d. Residuals vs. linear pred.
E. Response vs. fitted
C5
C6F
C6M

Fig. S6

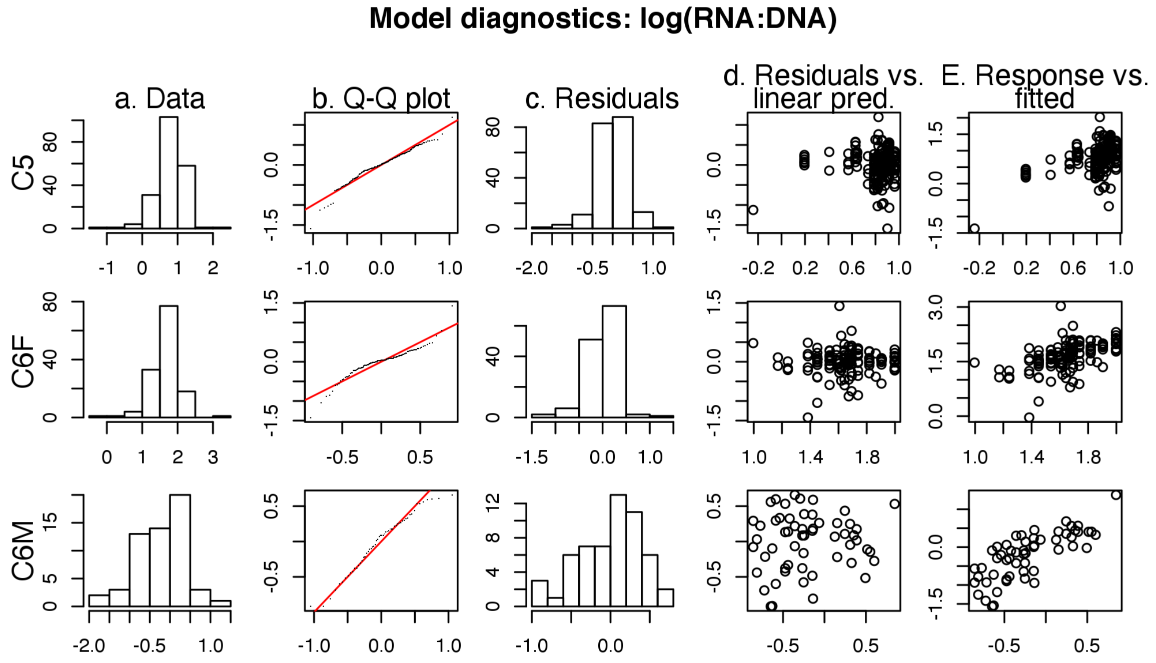
Model diagnostics: log(RNA:DNA)
a. Data
b. Q-Q plot
c. Residuals
d. Residuals vs. linear pred.
E. Response vs. fitted
C5
C6F
C6M

Fig. S7

Effects on C, N, DNA and RNA as µg or % of body weight

As for prosome area, lipid fullness, C:N and RNA:DNA (main text), we assessed effects of treatments on individual weights of C, N, RNA and DNA (µg or % of body weight). We lacked body weight measurements of copepods sampled for RNA:DNA. Therefore, to calculate %RNA and %DNA, we estimated body weight as a function of length using a length-weight regression based on copepods sampled for C:N (Fig. S8). The estimated relationship was:

$$\log_e(W) \sim -4.57 + 3.51 \times \log_e(L) \qquad \text{(S2)}$$

where W is body weight (mg) and L is prosome length (mm).

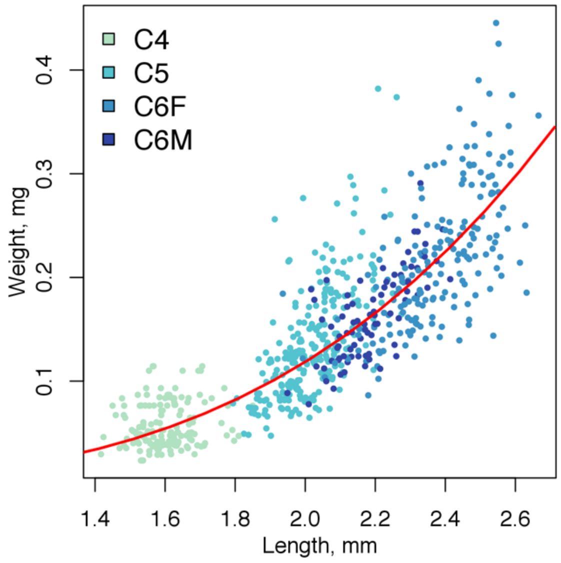

Fig. S8. Relationship between length (mm) and weight (mg) in *Calanus finmarchicus* copepods sampled for C:N analyses. Points: Observations with different colors indicating developmental stage. Red line: estimated length-weight regression (adjusted $R^2 = 0.77$).

We investigated effects of treatments on individual weights of C, N, RNA and DNA by fitting the following GAMM to data per developmental stage:

$$Y \sim \beta + F + P + g(D \times F) + g(D \times P) + g(T) + \varepsilon \qquad \text{(S3)}$$

Eq. S3 corresponds to Eq. 2 in the main text: Y is the response variable for a given stage, β is the intercept, F and P are factor variables of food and predator cue treatments (defined as high/low food, ±predator cues), $g(D \times F)$ and $g(D \times P)$ are interactions between food or predator cues and a smooth function of sampling day (with maximally 4 knots, i.e. 3 degrees of freedom), $g(T)$ is a random effect of experimental tank and ε is a normally distributed error term.

Results from Eq. S3 are given in Fig. S9 and Table S1. C and N (µg) responded positively to food and negatively to predator cues, reflecting results for prosome area (main text). For C6M, only the positive effect of food on N was significant. For C5, predator cues had a negative effect on %C and no effect on %N, while food had (non-significant) positive effect on %C and (non-significant) negative effect on %N, suggesting that results for C:N (main text) were driven by a negative effect of predator cues on C and a positive effect of food on C. In C6F, %C increased with food and decreased with predator cues, and %N increased with predator cues, suggesting that the positive effect of food on C:N was driven by increased C, and the negative effect of predator cues on C:N by a combination of reduced C and increased %N.

RNA:DNA was not significantly associated with food or predators cue in C5 (main text), but both DNA (µg) and RNA (µg and % of body weight) increased with food, and RNA (µg) decreased with predator cues. For C6F, DNA (µg) was negatively related to predator cues but unrelated to food, RNA (µg) was positively related to food and unrelated to predator cues, and %RNA was positively related to both food and predator cues (similarly to RNA:DNA). This explains the positive effects of both food (via increased RNA and %RNA) and predator cues (via reduced DNA and increased %RNA) on RNA:DNA. DNA as % of body weight did not differ between treatments in any stage.

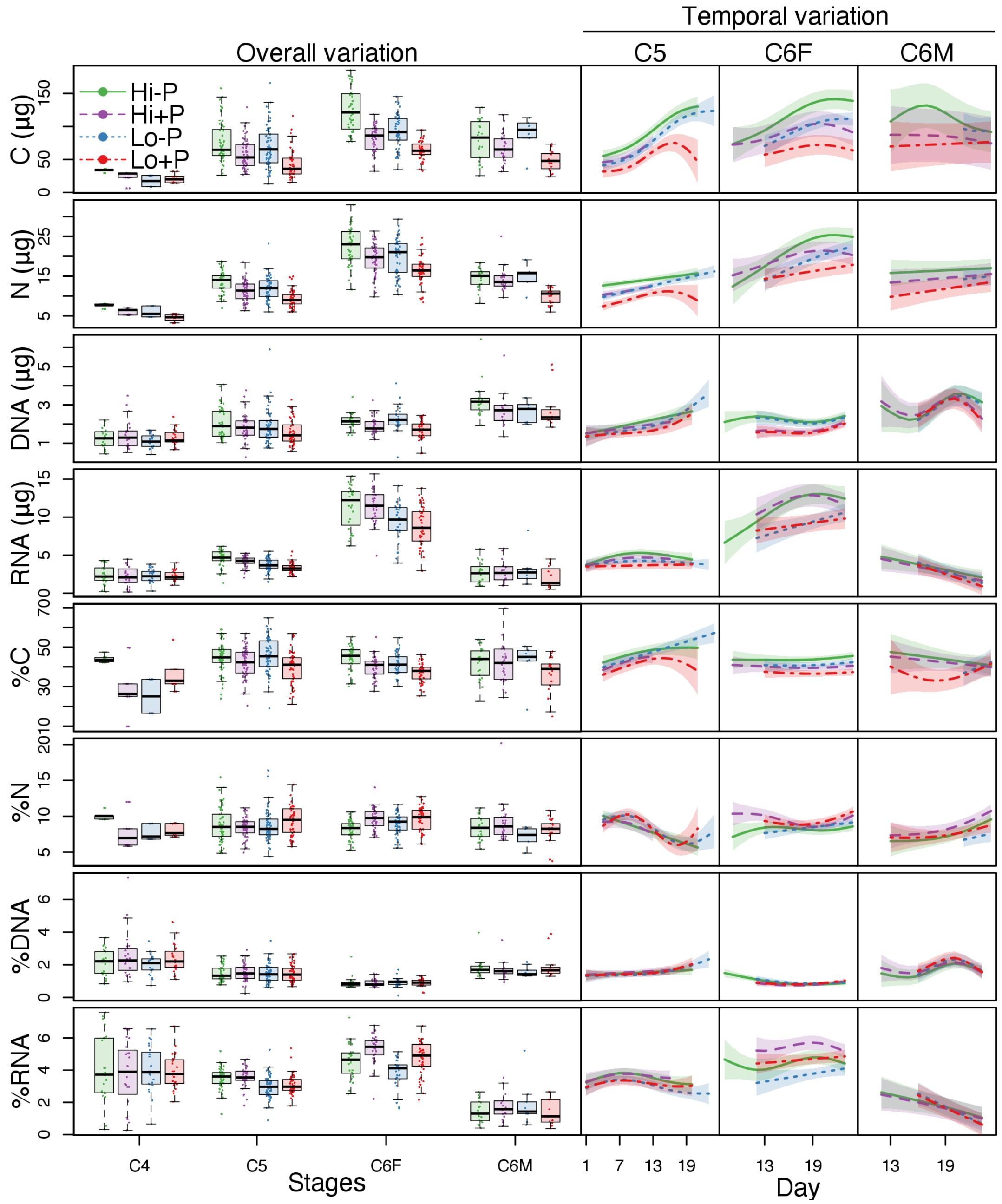

Fig. S9. *Calanus finmarchicus* C, N, RNA and DNA as individual weight (µg) or % of body weight per stage and treatment. Left panel: Observations per stage and treatment (points) with overlain boxplot showing the median (line), the interquartile range (box) and 1.5 × the interquartile range (whiskers) of the data. Right panels: Predicted temporal variation per stage and treatment from the statistical model (Eq. S3, colored lines), with 95% confidence interval of the prediction (shaded areas). Predictions are plotted for days with available data for the given stage and treatment (data were not sufficient to fit Eq. S3 for C4). The x-axis differs between stages. Hi-P: high food, no predator cues; Hi+P: high food, predator cues; Lo-P: low food, no predator cues; Lo+P: low food, predator cues.

Table S1. Adjusted R^2, estimates of model coefficients for parametric factor variables, and P-values for all terms in the statistical models with *Calanus finmarchicus* C, N, RNA and DNA (µg or % of body weight) per developmental stage as response variable (Eq. S3).

			Coefficient estimates		P-values						
								Day × Treatment			
		R^2	Food	Predator cue	Food	Predator cue	Tank	Low food	High food	No predator cue	Predator cue
C (µg)	C5	0.61	0.52	-0.60	**<0.01**	**<0.01**	0.14	0.14	0.07	**<0.01**	**<0.01**
	C6F	0.65	0.90	-1.11	**<0.01**	**<0.01**	**<0.01**	0.96	0.23	**<0.01**	0.36
	C6M	0.47	0.23	-0.99	0.65	0.06	**<0.01**	0.19	0.70	0.05	0.26
N (µg)	C5	0.40	0.68	-0.78	**<0.01**	**<0.01**	0.30	0.75	0.29	**<0.01**	**0.01**
	C6F	0.46	0.80	-0.68	**<0.01**	**<0.01**	**0.02**	**0.01**	**0.01**	**<0.01**	0.90
	C6M	0.38	0.79	-0.56	**0.01**	0.07	0.09	0.19	0.65	0.57	0.10
DNA (µg)	C5	0.19	0.30	-0.21	**0.02**	0.10	0.50	**0.03**	0.79	**<0.01**	0.09
	C6F	0.26	0.14	-0.84	0.39	**<0.01**	0.35	0.06	0.43	0.23	**0.02**
	C6M	0.26	0.20	-0.32	0.43	0.18	0.75	0.11	0.18	0.97	0.23
RNA (µg)	C5	0.42	0.88	-0.52	**<0.01**	**0.01**	**<0.01**	0.72	**0.04**	0.07	0.37
	C6F	0.35	1.00	-0.07	**<0.01**	0.72	0.06	0.10	**0.01**	0.05	0.97
	C6M	0.35	0.19	-0.25	0.46	0.33	0.24	**<0.01**	0.11	**0.02**	**0.01**
C (%)	C5	0.23	0.17	-0.51	0.19	**<0.01**	0.19	0.56	0.36	**0.02**	0.10
	C6F	0.18	0.50	-0.74	**<0.01**	**<0.01**	0.45	0.80	0.56	0.25	0.91
	C6M	0.09	0.41	-0.14	0.10	0.57	0.37	0.10	0.33	0.67	0.83
N (%)	C5	0.34	-0.28	-0.07	0.08	0.67	**0.03**	**<0.01**	0.28	0.13	**0.03**
	C6F	0.25	-0.20	0.70	0.15	**<0.01**	0.29	0.90	0.28	0.08	**0.01**
	C6M	0.32	0.61	0.50	**<0.01**	0.02	0.58	0.52	**0.01**	0.19	**0.04**
DNA (%)	C5	0.06	0.12	0.09	0.38	0.50	0.57	0.12	0.80	0.08	0.12
	C6F	0.21	-0.19	-0.04	0.24	0.83	0.80	0.08	**<0.01**	0.75	0.22
	C6M	0.26	-0.10	0.12	0.72	0.60	0.48	**0.01**	0.12	0.75	0.50
RNA (%)	C5	0.24	0.62	0.06	**<0.01**	0.75	**0.02**	0.17	0.19	0.24	0.38
	C6F	0.24	0.64	0.82	**<0.01**	**<0.01**	0.41	**0.02**	0.28	0.62	0.16
	C6M	0.45	0.03	-0.09	0.92	0.75	0.09	**<0.01**	0.25	**<0.01**	**0.01**

Notes: Coefficient estimates indicate the mean predicted change in the response variable, given as stage-specific standard deviations, when the predictor variable moves from low to high (food level) or from absence to presence (predator cue). Interactions between day and food or predator cue are formulated as different smooth effects of day under different factor levels. P-values < 0.05 are bolded.
